# Consistency and coherence in treatment outcome measures for borderline personality disorder

**DOI:** 10.1186/s40479-014-0022-5

**Published:** 2015-01-24

**Authors:** Alok Madan, J Christopher Fowler

**Affiliations:** The Menninger Clinic, 12301 South Main Street, Houston, TX 77035 USA; Department of Psychiatry & Behavioral Sciences, Baylor College of Medicine, Houston, TX USA

**Keywords:** Borderline personality disorder, Outcomes assessment, Treatment effectiveness

## Abstract

There is little consensus regarding outcomes assessment in borderline personality disorder treatment trials, making comparisons of results and meta-analytic studies difficult and far less generalizable. The current article highlights a range of measures frequently employed and puts forth a set of recommendations for a core battery of outcome measures in BPD treatment efforts. The proposed core battery aims to be comprehensive while minimizing patient burden, clinician time and costs. The relative brevity of the proposed core battery would engender flexibility for adding specific processes and outcome measures unique to targeted interventions and treatment models.

## Background

Treatment options for borderline personality disorder (BPD) have increased in recent decades. Psychotherapies including dialectical behavior therapy and mentalization-based therapy [1] with adjunctive pharmacotherapy targeting specific symptoms and/or comorbid psychiatric disorders [2] have demonstrated efficacy. There is international consensus regarding this evidence-based practice [3-5]. Future treatments likely will relieve further suffering; however treatment options for BPD are still in their relative infancy. As such, there is opportunity to build consensus regarding what constitutes an “effective treatment.” This dialogue is notably missing from the current BPD treatment literature. Furthermore, there is little overlap of outcome measures across trials, making comparisons of results and meta-analytic studies far less generalizable. The current article highlights a range of outcome measures assessed in treatment trials and puts forth a set of recommendations for a *core* battery of outcome measures in BPD treatment efforts.

## Main text

Recent meta-analyses and reviews highlight the variety of outcomes utilized to operationalize short-term outcomes. Similar to the heterogeneity in the diagnostic phenomenology of BPD, there, too, is significant heterogeneity in outcomes – both in terms of domains measured and in methods of measurement (e.g., self-report vs. interview). In their review of psychotherapy trials, Stoffers and colleagues [1] dichotomized outcomes as primary and secondary. Primary outcomes included: overall BPD severity and BPD symptom severity (i.e., anger, affective instability, chronic feelings of emptiness, impulsivity, suicidality, parasuicidality/self-harm, general interpersonal problems, avoidance of abandonment, identity disturbance, and dissociative/paranoid ideation). Secondary outcomes included: psychiatric comorbidity, general distress, global assessment of functioning, attrition/noncompliance with treatment, and adverse events. Additional outcomes included (in some studies) measurement of hospitalizations and emergency department visits. Note, however, that no study assessed all domains, and there was little overlap in measures used among the studies. Lieb et al.’s [2] review of pharmacotherapy trials included all of the aforementioned outcomes but also included medication tolerability and side-effects. There was a similar pattern of multiple domains assessed and multiple methods of measurement with little overlap across studies. Noting the lack of coherence and consistency across RCTs for BPD, Lieb and colleagues [2] as well as Zanarini and colleagues [6] both have called for a coherent set of measures to aid in systematic evaluation of treatment models and best practices.

Longer-term follow-up studies (e.g., [7-9]) suggest that many patients with BPD do well over time with most no longer meeting the required threshold number of diagnostic criteria; however, remission from a diagnosis and recovery from an illness are not comparable outcomes. With treatment and over time, some symptoms resolve (e.g., impulsivity) more readily than others (e.g., chronic dysphoria), but many individuals continue to have difficulties that require treatment [8,10,11]. These individuals will likely qualify for diagnosis of personality disorder, trait specified within the new DSM-5 system. Remission from a BPD diagnosis is a desired outcome but likely not the ultimate end point. The McLean Study of Adult Development and its extensions [9] define recovery as a constellation of symptomatic remission, good social and vocational functioning, and stability of the aggregate over an extended period of years. Recovery from BPD appears to be among the most stringent outcomes in the current literature; few treatment trials are likely to achieve this decades-long end point. Outside of formal research settings, assessment of recovery is rarely considered, much less done.

## Discussion

Based on broader experience with general mental health quality and outcomes efforts, we argue that a variety of additional outcomes may also define the efficacy of BPD treatment. Though infrequently and variably measured, quality of life (QOL) is gaining recognition as a primary treatment outcome, and the general consensus is that QOL is severely impaired in BPD but improves with treatment [12]. Of note, QOL is a necessary component for cost-effectiveness analyses, a growing area of significance in an era of healthcare cost containment efforts [13]. Measurement of QOL encompasses physical and somatic sensations in addition to other domains [14]. Specific measures of health-related functioning may also capture treatment outcomes in BPD given the increased prevalence of obesity and obesity-related illnesses [15], chronic pain [16], sleep disturbance [17], and increased use of medical care in general [18]. Finally, with the growing knowledge base of biomarkers of BPD, changes in inflammatory markers [19], neuropeptides [20], and/or neuroanatomical structures and functions such as the amygdala, hippocampus, and prefrontal cortex [21] are all promising outcomes of BPD treatments.

## Conclusions

It is not surprising that such a heterogeneous, debilitating, and stigmatized disorder would have such a varied use of outcomes across its empirical treatment base. Unfortunately, important outcomes are frequently excluded from BPD treatment trials, likely in an effort to reduce participant burden. Though psychotherapy is the only empirically supported treatment for BPD [3-5], few process variables (e.g., therapeutic relationship) are included as primary or secondary outcomes, despite their potential to affect treatment outcomes [22]. Furthermore, reductions in polypharmacy would also lend support to the effectiveness of an intervention – especially in light of increasing recognition that pharmacotherapy should only be used for targeted symptoms [2]. Inclusion of global assessments of disability, such as the World Health Organization’s Disability Assessment Schedule 2.0 (WHO-DAS 2.0), would put outcomes measurement in BPD in line with the updated Diagnostic and Statistical Manual of Mental Disorders, Fifth Edition (DSM-5) system, which uses scores from the WHO-DAS 2.0 to replace Global Assessment of Functioning (GAF) scores. Finally, assessment of patient satisfaction is routinely done across medical, surgical and psychiatric settings given its known relationship with compliance with treatment recommendations; this could also be included in a BPD outcomes battery. Note, measurement of patient satisfaction tends to be treatment setting specific (e.g., [23]) and thus would vary among trials.

There have been calls for a consensus to identify outcomes measures that capture quality of life, functioning and symptoms that are relevant to both patients and care providers (e.g. [4]); these have yet to be answered. We agree that inclusion of these core constructs is relevant and likely achievable in research and clinical settings; however, as highlighted above, the number of outcomes for BPD are extensive. Unfortunately, even if possible in formal research settings, a comprehensive battery is unlikely to be assimilated into busy clinical practices – despite the link between routine assessment of outcomes and improved treatment outcomes [24]. We are hopeful, however, that having an extensive but agreed upon battery of outcomes assessments will increase consistency across research settings and improve utilization within clinical practice. Based on broader experience with general mental health outcomes assessment in inpatient and outpatient psychiatric settings [23,25-27], we propose a self-report *core* battery in Table 1 that is comprehensive while minimizing patient burden, clinician time and costs. Each of these measures is commonly used in clinical and research settings, has sound psychometric properties, and provides a balanced but brief measurement of select underlying constructs. Many of the proposed measures have been translated in multiple languages and thus can be used globally. Of course, we encourage *supplementing* the core battery with additional self-report and interview-based measures as well as behavioral observation when possible. More in depth measurement will allow for a richer and potentially less-biased appreciation of treatment outcomes (e.g., measurement of changes in personality structure and/or adverse effects) but undoubtedly will be more time, labor and cost intensive.

**Table 1:** **Recommended core assessment battery for BPD effectiveness research**

**Domain**	**Measure**	**Relevant psychometric properties**
BPD Symptoms & Severity	Borderline Symptom Checklist [28]	Cronbach’s α = .97; 1 week test-retest reliability,
		r = .84; good divergent and convergent validity
Psychiatric comorbidity	Patient Health Questionnaire Somatic, Anxiety, and Depressive Symptom Scale [29]	Chronbach’s α = .80-.92; test-retest reliability,
		r = .83-.84; sensitivity = .66-.89; specificity = .71-.94
Substance Misuse	World Health Organization Alcohol, Smoking, and Substance Involvement Screening Test [30]	Cronbach’s α = .89; sensitivity = .80-.97; specificity = .71-.96; good divergent and convergent validity
Quality of Life	SF-12 [31]	Test-retest reliability, r = .76-.89; good divergent and convergent validity
Daily Functioning	World Health Organization Disability Assessment Schedule 2.0 [32]	Cronbach’s α = .94-.96; test-retest reliability, ICC = .93-.96; good construct and concurrent validity
Reduction in polypharmacy	% prescribed multiple antipsychotics	N/A; Note, however, this metric is in line with the Hospital-Based Inpatient Psychiatric Services Core Measures [33]
Service Utilization	Days hospitalized	N/A
Suicide related behaviors	Self-rated, electronic Columbia Suicide Severity Rating Scale [34,35]	Cronbach’s α = .73-94; Kappa: .79 – 1.00; acceptable sensitivity and specificity
